# Smart Wearable Sensors Based on Triboelectric Nanogenerator for Personal Healthcare Monitoring

**DOI:** 10.3390/mi12040352

**Published:** 2021-03-25

**Authors:** Ruonan Li, Xuelian Wei, Jiahui Xu, Junhuan Chen, Bin Li, Zhiyi Wu, Zhong Lin Wang

**Affiliations:** 1 School of Chemistry and Chemical Engineering, Guangxi University, Nanning 530004, China; liruonan0918@163.com (R.L.); binli@gxu.edu.cn (B.L.); 2 Center on Nano-Energy Research, School of Physical Science & Technology, Guangxi University, Nanning 530004, China; 3 Beijing Institute of Nanoenergy and Nanosystems, Chinese Academy of Sciences, Beijing 100083, China; weixuelian@binn.cas.cn (X.W.); xujiahui@binn.cas.cn (J.X.); chenjunhuan19@mails.ucas.ac.cn (J.C.); 4 College of Nanoscience and Technology, University of Chinese Academy of Science, Beijing 100049, China; 5 CUSPEA Institute of Technology, Wenzhou 325024, China; 6 School of Materials Science and Engineering, Georgia Institute of Technology, Atlanta, GA 30332, USA

**Keywords:** smart wearable sensor, triboelectric nanogenerator, health monitoring, fall-down alarm system

## Abstract

Accurate monitoring of motion and sleep states is critical for human health assessment, especially for a healthy life, early diagnosis of diseases, and medical care. In this work, a smart wearable sensor (SWS) based on a dual-channel triboelectric nanogenerator was presented for a real-time health monitoring system. The SWS can be worn on wrists, ankles, shoes, or other parts of the body and cloth, converting mechanical triggers into electrical output. By analyzing these signals, the SWS can precisely and constantly monitor and distinguish various motion states, including stepping, walking, running, and jumping. Based on the SWS, a fall-down alarm system and a sleep quality assessment system were constructed to provide personal healthcare monitoring and alert family members or doctors via communication devices. It is important for the healthy growth of the young and special patient groups, as well as for the health monitoring and medical care of the elderly and recovered patients. This work aimed to broaden the paths for remote biological movement status analysis and provide diversified perspectives for true-time and long-term health monitoring, simultaneously.

## 1. Introduction

Wearable devices play a great role in the acquisition and analysis of health information in recent years. It is an effective method for early detection, early diagnosis, and early treatment of diseases. For example, long-term monitoring of body signs such as electrocardiogram, heart sounds, blood pressure, and pulse is considered in the diagnosis and analysis of cardiovascular diseases [1]. However, the electricity required for wearable devices is mainly generated by traditional electrochemical batteries [2]. They do not only make wearable sensors bulky and uncomfortable to wear, but also requires frequent charging or regular replacement. Wearable energy harvesters, based on different principles, were widely developed, such as electrostatic [3,4], electromagnetic [5,6,7], thermoelectric [8,9,10], piezoelectric [11,12], and triboelectric [13,14]. As an energy harvester device, triboelectric nanogenerator (TENG) based on a coupling effect of triboelectrification and electrostatic induction was proved to be very effective to precisely convert mechanical energy (especially low frequency) in the environment into electricity [15,16,17,18]. The active sensor based on TENG also showed many advantages, for instance, extremely high sensitivity, strong adaptability, high flexibility, and good environment friendliness [2,19,20,21,22]. TENG was regarded to be a promising alternative to human mechanical energy harvesting [23,24,25,26,27,28,29,30] and self-powered active sensing [31,32,33,34,35,36,37,38,39].

Recently, combining biosensor technology, wireless communication technology, and life medicine knowledge to achieve real-time acquisition and monitoring of human health information is the main development direction of healthcare [40,41]. The wearable self-powered sensors based on TENG show excellent performance in many aspects, such as self-powered wireless transmission system [37] for real-time heart monitoring; self-powered pressure sensor [42] as a blood pressure detector to achieve the prevention and diagnosis of cardiovascular disease; electronic-skin devices [43] present an accurate measurement for monitoring wrist pulse; smart textiles [44,45] for sleep monitoring and evaluation; wearable sensors [22,46] that are conformally attached to human skin to monitor limb and finger movement; and TENG-based wearable exercise system for upper limb rehabilitation post neurological injuries [47]. Excellent output and sensing performance are always shown in these sensors. It would be significant to human health to design a sensor that does not have limited application time, can be flexibly worn in any place of the body or clothing, and is comprehensive for detecting basic human survival movements like walking, running, and sleeping.

Here, we designed a self-powered smart wearable sensor (SWS) consisting of PTFE and Fe, which can be worn as a hand ring or foot ring, and can also be glued to the body or cloth to meet different needs. Due to its strong electronic attraction, PTFE is easy to gain triboelectric charges from almost any other materials [48]. This behavior is attributed to the large amount of fluorine in PTFE, which has the highest electronegativity among almost all elements [49]. Steel is at a more positive direction than wood, nickel, and copper in the triboelectric sequence [50]. As a result, PTFE and Fe become negatively and positively charged after contact, respectively. The created SWS only needs cheap and easy-to-obtain raw materials and a simple preparation process, which greatly improves the mechanized production. The SWS shows nearly no attenuation of electrical output performance during 1000 cycles, showing excellent stability and mechanical durability. In addition, the SWS exhibits a fast response time of less than 100 ms, which would facilitate the real-time monitoring of health. Based upon the output amplitude and the response time between the peak and valley, different motion states of stepping, walking, running, and jumping could be monitored and identified. More importantly, the SWS could immediately detect the event of an accidental fall and send a call out to the family or doctor in time. Concurrently, sleep monitoring and early warning of potential respiratory diseases could also be performed, which could be used for early diagnosis of heart/respiratory diseases. The SWS has many advantages of small size, light weight, comfortable wearability, excellent stability, mechanical durability, rapid response, real-time monitoring, accurate identification, and timely warning. It is especially not limited by time and place and can be worn anywhere on the body or cloth, which provides a more convenient choice for human motion status monitoring and health evaluation, and can be widely used in motion tracking and medical care systems.

## 2. Materials and Methods

### 2.1. Fabrication of the SWS

Two acrylic sheets were first processed by laser cutting technology [51] with a laser cutter (6090, 31 Degree Technology, Jiaxing, China) to form a ring with 16 mm in the inner diameter, 20 mm in the outer diameter, and 10 mm in thickness, and two round platforms with 10 mm in diameter and 1 mm in thickness. Then, a pair of through circular holes of 1 mm in diameter were made on the ring and the center of the round platforms, respectively. The Cu paste (≈30 μm after drying), evenly coated on the inner diameter of the ring with two blank gaps made between the pair of holes and the opposite side, were also applied to the center of the round platforms with a diameter of 6 mm to form electrodes, respectively. Subsequently, the original commercial 50 μm thickness PTFE film with a special nanopatterning procedure was used as the dielectric layer on the electrode layers. An Fe ball with a diameter of 6 mm was used as the triboelectric layer material. In particular, two thin rings with an inner diameter of 12 mm, an outer diameter of 14 mm, and a thickness of 1 mm were designed and pasted on the round platforms as barriers, to prevent the ball from contacting the round platforms and the ring simultaneously. All parts were assembled with adhesive, and wires were connected with the soldering tin at the holes for measurement.

### 2.2. Characterization and Measurement

Scanning electron microscopy (SEM, SU8020, Hitachi, Tokyo, Japan) was used to characterize the surface morphology of the PTFE film. To systematically investigate the output performance of the SWS, a linear motor (E1200, LinMot, Spreitenbach, Switzerland) was utilized to simulate the press-release. Open-circuit voltage (*V*_OC_) was measured through multi-channel data acquisition, processing, and analysis, which were available on LabVIEW (National Instruments, Cleveland, OH, USA). The health monitoring system consisted of the SWS, maintenance communications unit (MCU) (Arduino uno r3), Bluetooth transmitter (HC-08), Bluetooth receiver (a phone), and analysis software. 

## 3. Results and Discussions

### 3.1. Preparation and Principle of the SWS

**Figure 1:**
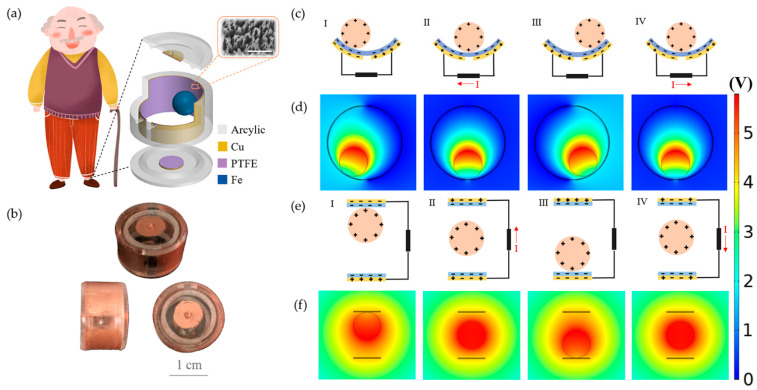
Overview of the SWS for healthcare monitoring. (**a**) The SWS was attached to the shoe to serve as healthcare monitoring. The illustration shows the SEM image of the PTFE film. The scale bar is 1 μm. (**b**) Physical pictures of the SWS. The scale bar is 1 cm. (**c**) Working mechanism of the SC. (**d**) The voltage distribution between the Fe ball and PTFE film with Fe ball moving along the circumference. (**e**) Schematic diagrams of the TBC. (**f**) The voltage distribution between the Fe ball and the PTFE film with the Fe ball jumping up and down.

Figure 1a shows a monitoring system of the SWS wore on the shoe. On the right was an enlarged schematic diagram of the SWS. To make the monitoring signal comprehensive and accurate, we produced a dual-channel SWS. The SWS consisted of a side channel (SC) adhered on the inner side of the acrylic circular ring, a top and bottom channel (TBC) placed on two acrylic covers, and an Fe ball rotating in the cavity formed by the acrylic shell. The inset was an SEM image of the PTFE film. The topography of the PTFE layer presented nanowires. Figure 1b displayed entities of the SWS in different directions. The detailed fabrication process is discussed in the Materials and Methods. The working principle of the SC is based on the coupling effect of triboelectrification and electrostatic induction, as shown in Figure 1c. The Fe ball is positively charged because of contact with the PTFE film and can be stored for a long time. The movement of the Fe ball on the dielectric layer (PTFE film) caused the Cu electrodes under the PTFE film to produce an uneven charge distribution, resulting in the transfer of electrons between the two electrodes to balance local potential distribution. Therefore, a current could be generated between the electrodes, which corresponded to the motion states of the Fe ball. The specific performance was presented as follows. When the Fe ball was in contact with the PTFE film, the electrons were transferred from the surface of the Fe ball to the surface of the PTFE film on account of the difference in electron affinity between the two. As a result, the PTFE film was negatively charged and the Fe ball was positively charged. The triboelectric charge could not be conducted or neutralized for a period. At this time, the positive friction charge was fully compensated by the opposite friction charge, so no electrical output was generated on the electrode (Figure 1c(I)). Once the Fe ball moved to the right, the balance of the electric field was broken, and the potential difference drove the electrons on the left electrode to move to the right electrode, which generated a leftward current (Figure 1c(II)) that balanced the potential until a new electrical balance was established (the Fe ball moved to the rightmost end, Figure 1c(III)). Similarly, when the Fe ball moved to the left, the potential difference drove the flow of electrons to generate a rightward current (Figure 1c(IV)) until the initial state. The potential distribution between the Fe ball and PTFE film with the Fe ball moving from right to left could also be simulated using COMSOL, as depicted in Figure 1d. The COMSOL simulation animation of the SC is shown in Video S1. Meanwhile, the working mechanism diagram and potential distribution simulation diagram of the TBC is in Figure 1e,f. The COMSOL simulation animation of the TBC is illustrated in Video S2. The two channels were basically similar.

### 3.2. Electrical Performance of the SWS

**Figure 2:**
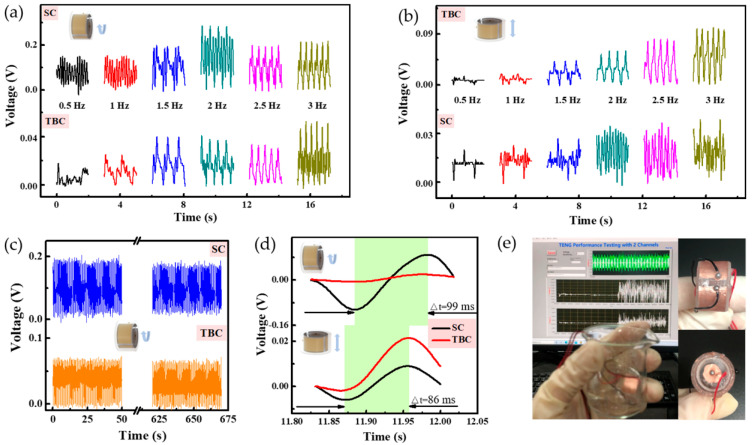
Electrical and mechanical characterization of the SWS. *V*_OC_ of the SWS at various pressing frequencies in different moving directions. (**a**) Circumference direction; and (**b**) top and bottom direction. (**c**) The mechanical durability characterization of the SWS with 1000 continuous working cycles at 1.5 Hz. (**d**) Response time of the *V*_OC_ when the external stimulus was applied and released at the acceleration of 1.7 m/s^2^. (**e**) Water resistance of the SWS.

Experimental setup for the fundamental test is presented in Figure S1. First, the response of the SWS to various pressing frequencies was explored. Previous studies of examined energy of particular human motions indicate that human motion is a combination of low frequency vibrations (≤10 Hz), the dominant motion frequency range was 1.1–3.8 Hz [52]. The *V*_OC_ of two channels of the SWS in the frequency range of 0.5 Hz to 3 Hz moving in the circumference direction, and the top and bottom direction is demonstrated in Figure 2a,b, respectively. The influence of noise was distinctive when voltage data were very small at 0.5 Hz and 1 Hz. The voltage increased with the increase of the frequency, and the noise was less obvious. The peaks for higher frequencies were clearer. Simultaneously, with the increasing of the frequency, the *V*_OC_ first increased and then decreased when rotating along the circumference, while the *V*_OC_ continuously increased when jumping top and bottom. It was likely that a poor contact between the Fe ball and the side existed at a faster frequency. Therefore, it looked like there was no outstanding linear output voltage depending on the frequency. The output of the SC was larger than TBC, when moving in a circumference direction but was opposite in the top and down direction. It was mainly affected by the direction of the applied force, the channel in the direction of the applied force would be larger than the other channel. The *V*_OC_ of two channels of the SWS when rotating along the circumference was slightly larger than that jumping top and bottom, because the contact area of the dielectric layer of the SC was bigger than that of the TBC, which was also consistent with the basic principle of TENG.

To summarize the influence of the triboelectric materials, number of channels, and moving direction on the basic performance of the SWS, the basic electrical outputs of side-channel Fe-triboelectric SWS (FT-SWS) (Figure S2a) and side-channel PA-triboelectric SWS (PT-SWS) (Figure S2b) moving in the circumference direction; dual-channel PT-SWS moving in circumference direction (Figure S2c), and top and down direction (Figure S2d) were researched separately. The *V*_OC_ of two-channel PT-SWS rotating along the circumference fit better with the changing trend, while the SC of two-channel PT-SWS could not follow the changing trend well when it jumped up and down. This was perhaps owing to the lighter weight of the PA ball. In summary, the dual-channel FT-SWS had preferable sensitivity and practicality.

To discuss the mechanical properties of the SWS, the durability of the SWS was characterized by 1000 cycles at 1.5 Hz, represented in Figure 2c. The inset was SEM images after 1000 cycles, we could see that there was no obvious abrasion. It indicated the excellent stability, durability, and wear resistance of the sensor. As can be seen in Figure 2d, the response time of the SWS to the external load was taken care of. When the acceleration was about 1.7 m/s^2^, the response times of the two channels moving in two directions were less than 100 ms. Rapid response made sure that the SWS could catch the signals in time, under the action of the external stimulus. For verifying the credible of the SWS in wet environments like sports sweating, rain, snow, and other extreme weather, the water resistance of the SWS in a beaker containing water was tested, as illustrated in Figure 2e. The SWS was coated with a thin layer of silica gel to achieve the dual effects of protecting the electrodes and waterproofing simultaneously, as shown in Video S3. These results demonstrated that the SWS had the advantages of high sensitivity to external stimulus, long cycle stability, short response time, and excellent water resistance, which promoted the great potential of the SWS in health monitoring.

### 3.3. Monitoring of Motion State

**Figure 3:**
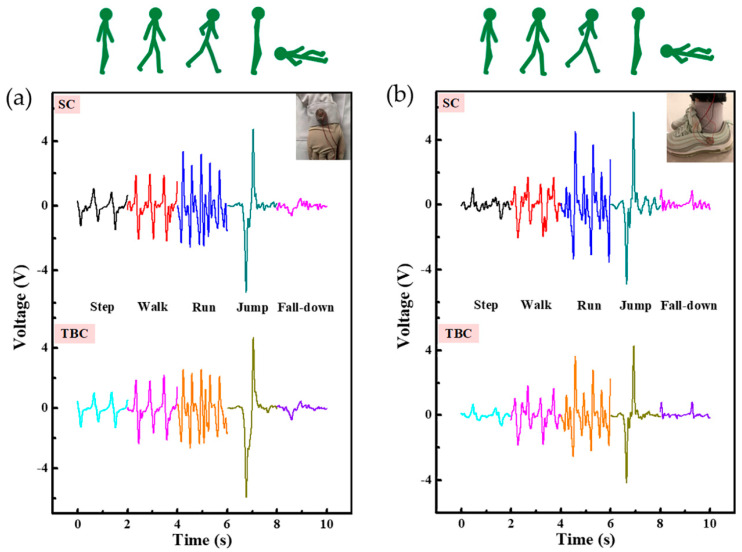
Motion state monitoring signals (Step, Walk, Run, Jump, Fall-down) of the SWS in different wearing parts—(**a**) wrist and (**b**) shoe.

Due to the convenient and comfortable wearability of the SWS, it could be placed on different parts of the human body. Figure 3a displays the signals of the SWS when it was laid on the wrist. When the wrist swung forward, a valley could be detected; when the wrist swung back, a peak could be detected. The amplitude and response time under each motion state were different. As the state of motion became more intense from stepping, walking to running, the amplitude increased and the response time decreased. Moreover, the amplitude of the SC was slightly larger than TBC, thanks to the smaller gap on the side. The amplitude of jumping was the largest, which probably related to the acceleration of gravity. The amplitude of the fall-down was the smallest. Similarly, Figure 3b shows output changes under different motion states where the SWS was placed on the side of the shoe to monitor the movement state. When the shoe stepped on the floor, a valley could be detected, when the shoe left the floor, a peak could be detected. The amplitude and response time corresponding to each movement state was the same as that worn on the wrist. Additionally, the voltage was slightly larger, which was possibly a result of the greater external stimulus exerted on the shoe. The amplitude and the response time were key factors for monitoring different exercise states.

Equally, in order to summarize the influence of the number of channels, triboelectric materials, and wearing parts on the SWS in actual application scenarios, the signals of the single-channel FT-SWS (Figure S3a), PT-SWS (Figure S3b) when they were on the wrist and shoe, two-channel PT-SWS on wrist (Figure S4a) and shoe (Figure S4b) were researched, respectively. From stepping, walking, running, jumping to falling down, the peak shape, amplitude, and response time of each movement state was recognizable but not so obvious. Monitoring the motion state, especially fall-down state, we needed the output of the SC and TBC simultaneously. Signals of the two-channel PT-SWS were a little bit smaller than that the FT-SWS, owing to the weight of the ball. Hence, the two-channel FT-SWS was chosen to conduct the next experiments and explorations. Moreover, the SWS could easily identify the state of motion, as well as expand its application in motion tracking, activity recognition, sports training, and other fields. For example, the travel distance (s) and motion speed (v) could be obtained by the equation expressed as Note S1.

### 3.4. Fall-Down Alarm System and Sleep Monitoring

**Figure 4:**
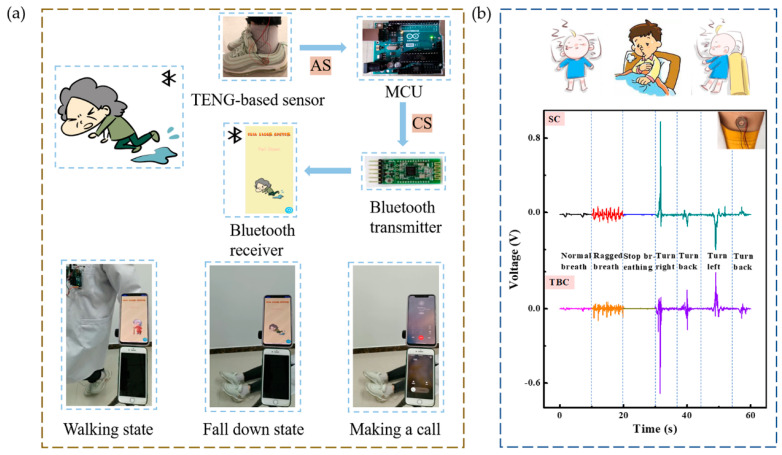
Demonstrations of the SWS for (**a**) motion state monitoring and fall-down alarm system and (**b**) sleep state monitoring.

To demonstrate that the SWS can serve as a sensing device for actual scenarios, a real-time health monitoring system was invented. The system is constructed of SWS, MCU, Bluetooth transmitter, Bluetooth receiver, and an analysis software, which is expressed in Figure 4a. The system could be used as a remote fall-down alarm system for the elderly and patients. To demonstrate this function, an SWS was placed on the side of the shoe. Different output signals are generated when users put on the shoe with the SWS for different sports, as shown in Figure 3b. By comparing the amplitude of the analogy signals (AS) of SWS, the MCU could process and provide a control signal (CS) ‘0′ or ‘1′. Then, the signal was sent by the Bluetooth transmitter to a Bluetooth receiver, as a notification. Figure 4a illustrates in detail. (I) When the user walks, the SWS continuously generates electric output, MCU indicates ‘0′, and the phone displays the status as “walking”. (II) If the user falls down on the ground, the SWS does not produce electrical output, MCU indicates ‘1′, the phone changes the status to “fall down” and activates an alert, meanwhile the app drives the phone to make a call to the added relatives or family doctors in a few seconds. This process is also shown in Video S4. It needs to be noted that so far no false alarm in the health monitoring of healthy people was observed, but it had a possibility of false alarm in the later monitoring of actual disabled people and the elderly. With the emergence of above problem, we will further improve the system.

The SWS was also placed on the neck to monitor the sleep state in actual time, as displayed in Figure 4b. When the user lay flat on the bed and breathed normally, the amplitude was small and response time was long. During sleep, breaths become shorter, the amplitude increases and the response time decreases. The amplitude and response time are almost 0, when sleep becomes terrible, and sleep apnea syndrome attacks. Individual health monitoring and medical care can be carried out according to the differences of amplitude and response time. During occurrence of tachypnea/dyspnea, inability of self-regulation (output signal shortness), obstructive apnea (output signal disappearance), the monitoring system can timely remind family members/doctors. This system can be used for early diagnosis of heart/respiratory diseases.

When the wearer turns over to the right, it can be observed that there is a peak in the SC and a valley in the TBC. When turning back from right to left, the SC has a valley, and the TBC has peak and valley. Interestingly, when turning left and turning back from left to right, the peak shape is just the opposite of the above. There is one output for each turn, and the peak shape of left and right turns are different. Family members can judge whether the distance from the bedside is dangerous according to the peak shape and the number of turns. It is significant for the healthy growth of babies and attention deficit hyperactivity disorder patients, and healthcare of elderly or patients with unconsciousness. All these applications demonstrate the potential of the SWS in self-powered intelligent sensor systems.

## 4. Conclusions

In summary, a wearable and practical smart sensor based on two-channel TENG is demonstrated for true-time health monitoring. The SWS have many outstanding advantages, including simple manufacturing, low cost, convenient wearability, high durability, short response time, and excellent water resistance, which is not limited by time and place and can be worn anywhere on the body or cloth, according to needs. The SWS can not only realize real-time health monitoring, but is also suitable for mass production. In addition, the designed SWS can distinguish different motion states, such as stepping, walking, running, jumping, and can calculate movement distance and speed at the same time. Meanwhile the SWS can be used as an alarm system for a fall-down event, sleep breathing diseases and sleep safety, which is of consequence to babies, the elderly, and potential patients. This research proposes a new design scheme for wearable motion monitoring equipment, which can be further applied to motion recognition, remote medical care monitoring, and other health assessments.

## Data Availability

The data presented in this study are available from all authors.

## Supplementary Materials

The following are available online at https://www.mdpi.com/2072-666X/12/4/352/s1. Figure S1: Experimental setup for the fundamental test. Figure S2: Electrical characterization of the SWS with different triboelectric materials, number of channels and moving directions—side-channel (a) FT-SWS, and (b) PT-SWS rotating along the circumference; dual-channel PT-SWS moving in (c) circumference direction and (d) top and down direction. Figure S3: Motion state monitoring signals of side-channel (a) FT-SWS and (b) PT-SWS when they were put on the wrist and shoe. Figure S4: Motion state monitoring signals of two-channel PT-SWS in different wearing parts—(a) wrist and (b) shoe. Note S1. The equation of travel distance and motion speed obtained by the sensor. Video S1. The COMSOL simulation animation of SC. Video S2. The COMSOL simulation animation of TBC. Video S3: Water resistance of the SWS. Video S4: Fall-down alarm system based on the SWS.
