# Supplementary material for: Smart Wearable Sensors Based on Triboelectric Nanogenerator for Personal Healthcare Monitoring
Source: Micromachines (Basel). 2021 Mar 25;12(4):352. doi: 10.3390/mi12040352 (PMC8064435; doi:10.3390/mi12040352)
Supplement: Supplementary file 1 [file micromachines-12-00352-s001.zip › micromachines-1155769-supplementary/micromachines-1155769-supplementary.pdf]

micromachines

Supplementary Materials: Smart Wearable Sensors Based on Triboelectric Nanogenerator for Personal Healthcare Monitoring

Ruonan Li, Xuelian Wei, Jiahui Xu, Junhuan Chen, Bin Li, Zhiyi Wu and Zhong Lin Wang

Supplementary Figures

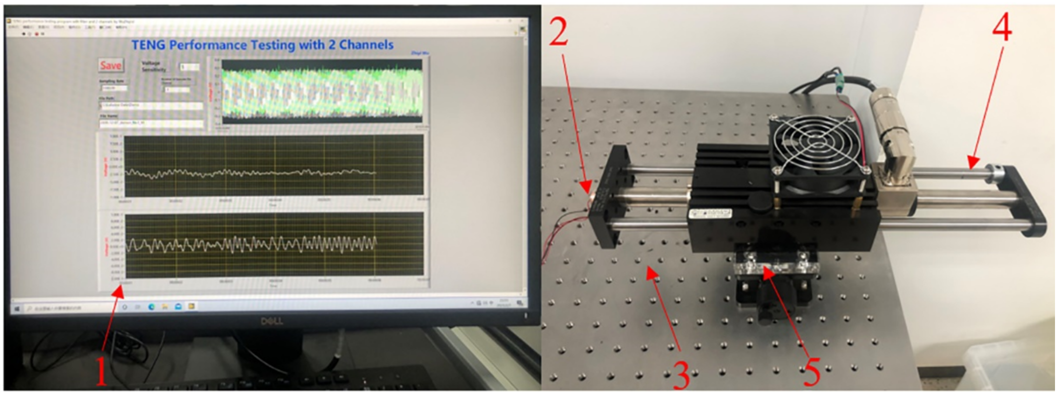

1. LabVIEW test program
2. The SWS
3. Optical table
4. Linear motor
5. Lifting platform

Figure S1. Experimental setup for the fundamental test.

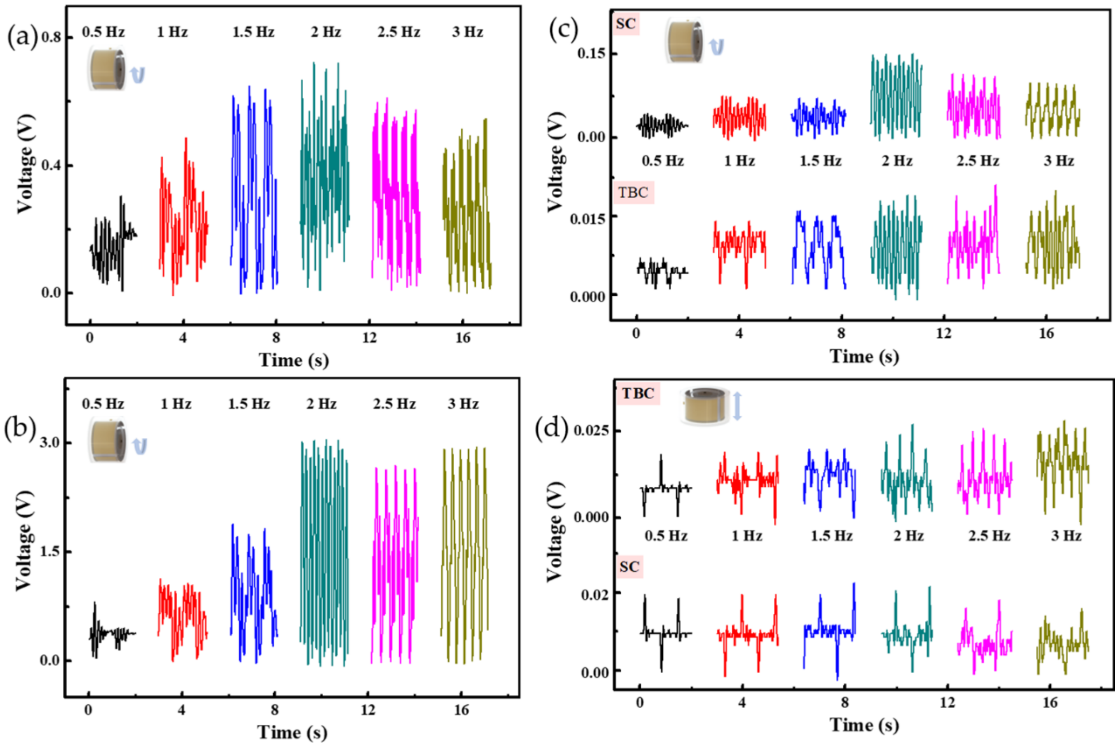

Figure S2. Electrical characterization of the SWS with different triboelectric materials, number of channels and moving directions: side-channel**(a)** FT-SWS and **(b)** PT-SWS rotating along the circumference; dual-channel PT-SWS moving in **(c)** circumference direction and **(d)** top and down direction.

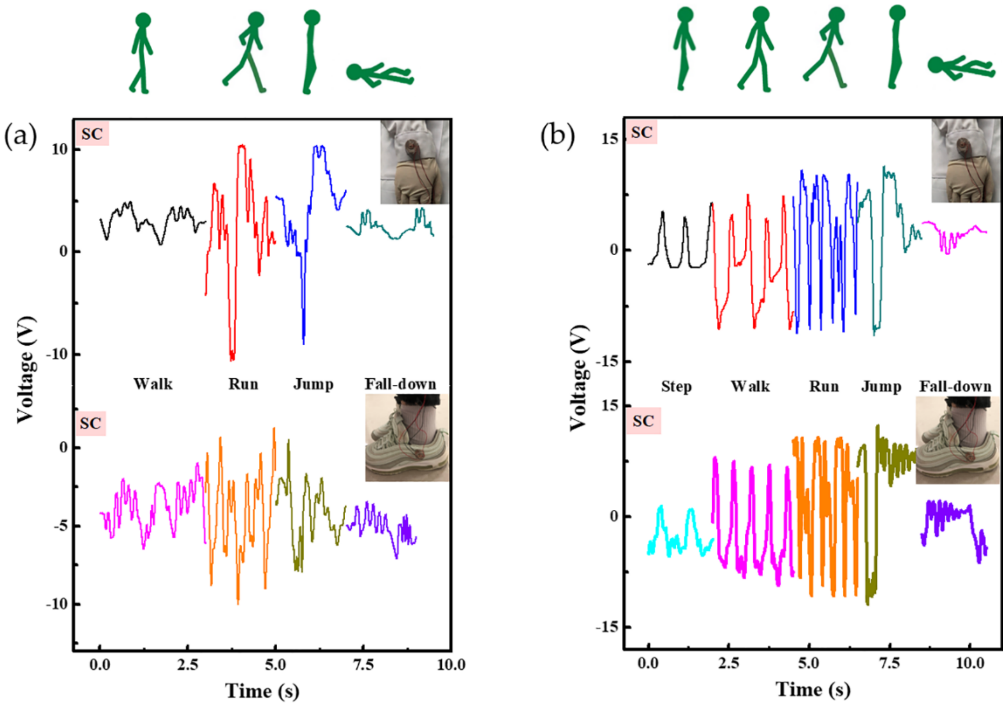

Figure S3. Motion state monitoring signals of side-channel **(a)** FT-SWS and **(b)** PT-SWS When they were put on the wrist and shoe.

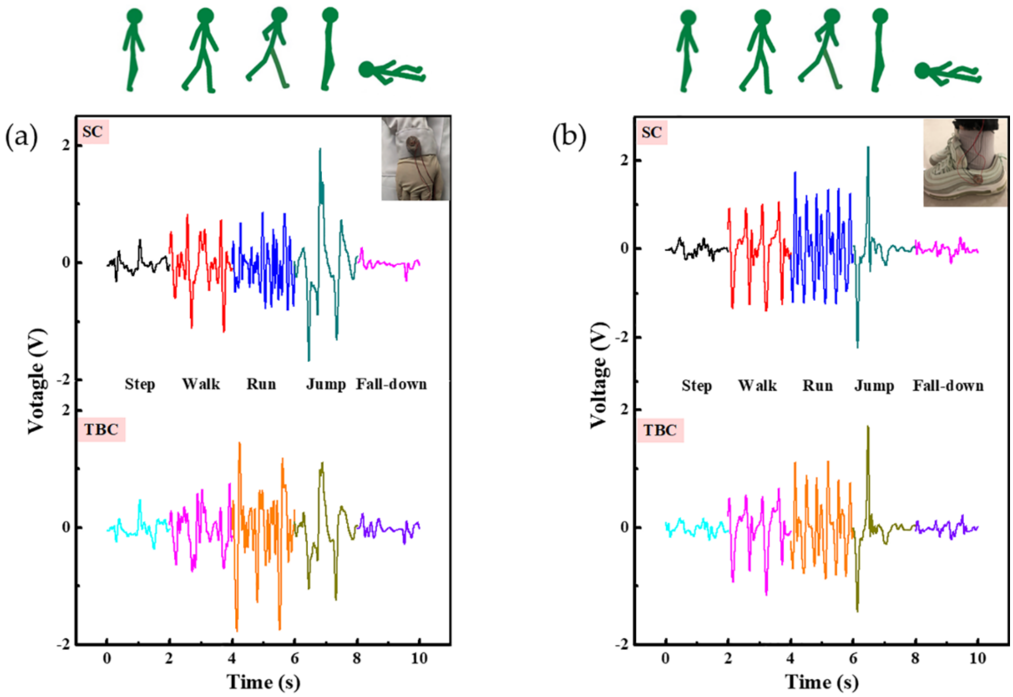

Figure S4. Motion state monitoring signals of two-channel PT-SWS in different wearing parts: **(a)** Wrist; **(b)** Shoe.

Supplementary Note

Note S1. The equation of travel distance and motion speed obtained by the sensor.

Movement distance (s) and motion speed (v) can be obtained by the following equations.

$$s = n \times L \tag{1}$$

$$v = L \times f \tag{2}$$

where L and f represent stride and frequency respectively, and n represents the number of positive or negative voltage peak.

Supplementary Videos

Video S1. The COMSOL simulation animation of SC.
Video S2. The COMSOL simulation animation of TBC.
Video S3. Water resistance rest of the SWS.
Video S4. The fall-down alarm system demonstration based on the SWS.
